# Identification of Histological Patterns in Clinically Affected and Unaffected Palm Regions in Dupuytren's Disease

**DOI:** 10.1371/journal.pone.0112457

**Published:** 2014-11-07

**Authors:** Camilo-Andrés Alfonso-Rodríguez, Ingrid Garzón, Juan Garrido-Gómez, Ana-Celeste-Ximenes Oliveira, Miguel-Ángel Martín-Piedra, Giuseppe Scionti, Víctor Carriel, Pedro Hernández-Cortés, Antonio Campos, Miguel Alaminos

**Affiliations:** 1 Department of Histology (Tissue Engineering Group), University of Granada, Granada, Spain, and Instituto de Investigación Biosanitaria ibs, Granada, Spain; 2 Ph.D. Programme in Biomedicine, University of Granada, Granada, Spain; 3 Division of Trauma and Orthopedic surgery, University Hospital San Cecilio, Granada, Spain; A*STAR, Singapore

## Abstract

Dupuytren's disease is a fibro-proliferative disease characterized by a disorder of the extracellular matrix (ECM) and high myofibroblast proliferation. However, studies failed to determine if the whole palm fascia is affected by the disease. The objective of this study was to analyze several components of the extracellular matrix of three types of tissues—Dupuytren's diseased contracture cords (DDC), palmar fascia clinically unaffected by Dupuytren's disease contracture (NPF), and normal forehand fascia (NFF). Histological analysis, quantification of cells recultured from each type of tissue, mRNA microarrays and immunohistochemistry for smooth muscle actin (SMA), fibrillar ECM components and non-fibrillar ECM components were carried out. The results showed that DDC samples had abundant fibrosis with reticular fibers and few elastic fibers, high cell proliferation and myofibroblasts, laminin and glycoproteins, whereas NFF did not show any of these findings. Interestingly, NPF tissues had more cells showing myofibroblasts differentiation and more collagen and reticular fibers, laminin and glycoproteins than NFF, although at lower level than DDC, with similar elastic fibers than DDC. Immunohistochemical expression of decorin was high in DDC, whereas versican was highly expressed NFF, with no differences for aggrecan. Cluster analysis revealed that the global expression profile of NPF was very similar to DDC, and reculturing methods showed that cells corresponding to DDC tissues proliferated more actively than NPF, and NPF more actively than NFF. All these results suggest that NPF tissues may be affected, and that a modification of the therapeutic approach used for the treatment of Dupuytren's disease should be considered.

## Introduction

Dupuytren's disease (DD) is a proliferative disorder affecting the palm of the hands that is characterized by an alteration of the cells and extracellular matrix (ECM) of the palm fascia. This alteration may lead to an irreducible and progressively disabling flexion and contracture of the fingers, with loss of function and deformity of the hand [1]. DD is a multifactorial disease, and several studies previously demonstrated the important role of genetics, alcohol, tobacco [2] and different systemic diseases such as diabetes, epilepsy and hyperlipidemia [3].

One of the main factors involved in the development of this disease is the proliferation of myofibroblasts in the affected tissues. Myofibroblasts share characteristics of both fibroblasts and smooth-muscle cells [4], and they may be the responsible for the tissue contracture found at the initial phases of DD [5]. In turn, the ECM usually has important alterations of both its fibrillar and non fibrillar components [2]. Although a comprehensive histological and genetic analysis of the fibrillar and non-fibrillar components of the ECM and the normal palm fascia has not been performed to the date, previous studies have identified alterations of type I and type III collagens, fibronectin, laminin and other ECM components in DD [6], along with an important disregulation of several genes encoding proteins in the WNT-signaling pathway [7].

The treatment of DD is complex, and it involves surgical and non-surgical approaches [8], [9], all of them with a unique goal of eliminate the affected tissue [9]. Non-surgical treatments are mainly based on the use of radiotherapy, physiotherapy, dimethylsulfoxide solutions and *Clostridium histolyticum* collagenase injections [10], [11]. However, the most effective treatments are the surgical removal of the fibrous cords causing the patient's symptoms by fasciectomy or fasciotomy [8], [9]. The risk of treatment failure and disease recurrence ranges between 8% and 66%, making necessary additional research on the causes and factors related to this recurrence, including treatment alternatives improving short and long-term outcome of DD patients.

Despite recent advances in understanding the pathophysiology of DD, the therapeutic approach is palliative and not curative [12]. In most cases, evolution of DD is progressive and irreversible, and the risk of relapse after surgical excision is high [2]. A better knowledge of the factors and mechanisms involved in the disease onset and progression not only in DD cords, but also in the rest of the hand fascia, whose role in this disease should be clarified, could contribute to a better treatment and prevention of postsurgical relapse. Typically, the disease only affects the central zone of the palmar aponeurosis [13] with the formation of a fibrous cord attached to the base of the middle phalanx and often the tendon sheath [12]. Other areas of the palmar fascia usually remain asymptomatic. However, studies failed to determine whether these areas are involved in the genesis and development of the DD and may influence the final outcome in this disease.

To shed light on this issue, in the present study we have carried out a complete analysis of both the cellular and ECM components of Dupuytren's disease contracture cords and palmar fascia clinically non-affected by Dupuytren's disease contracture by using histological, histochemical, immunohistochemical, gene expression and cell culture methods and techniques.

## Materials and Methods

### Tissue samples

In this work, we analyzed three types of tissues: Dupuytren's disease contracture cords (DDC); palmar fascia clinically unaffected by Dupuytren's disease contracture (NPF); and normal forehand fascia (NFF). The three tissue types were obtained from DD patients subjected to surgical removal of the DDC at the trauma and orthopedic surgery unit of the San Cecilio University Hospital of Granada (Spain) (n = 6 samples). All patients included in the study were male and their age ranged between 60 and 66 years. They all had severe chronic Dupuytren's disease with the presence of an evident DDC that compromised the movement of one of the fingers. None of the patients had been operated before. In each case, the size of the excised tissue was 1×1 cm. Immediately after removal, tissues were divided in three fragments. One of the pieces was fixed in 10% buffered formalin, dehydrated and embedded in paraffin for histological analysis. The second piece was used for mRNA isolation for microarray analysis. The last fragment was used for recultivation and cell proliferation experiments.

### Ethics Approval

In this study, each patient signed an Informed consent. This study was approved by the ethics committee from University of Granada, Granada, Spain.

### Histological and histochemical analyses

Sections of 5 mm-thickness were obtained from tissues embedded in paraffin by using a microtome. After dewaxing in xylene, washing in ethanol series and rehydrating in water, sections were processed as shown below. All samples were processed simultaneously.

1. For histological analysis of tissue structure, tissue sections were stained with Masson's trichrome staining method. Briefly, samples were incubated in solution A –0.5 ml acid fuchsin, 0.5 ml glacial acetic acid and 99 ml distilled water- for 15minutes, in solution B -1 g phosphomolybdic acid and 100 ml distilled water- for 10 minutes and in solution C – 2 g methyl blue dye, 2.5 ml glacial acetic acid and distilled water up to 100 ml- for 5 minutes. Then, samples were washed in distilled water, dehydrated in alcohol and xylene and mounted for light microscopy analysis.

2. To determine the number of cells per area of tissue (cell density analysis), tissue sections were stained with 4,6-diamidino-2-phenylindole (DAPI) and analyzed using a light microscope. All cell nuclei were automatically quantified using the Image J software.

3. To analyze the fibrillar components of the ECM by histochemistry, samples were stained as follows [14]:

– To evaluate the presence of collagen fibers, tissues were stained with the Picrosirius method using Sirius red F3B reagent for 30 min and counterstained with Harris' Hematoxylin for 5 min. To analyze the three-dimensional collagen fiber organization, samples stained with Picrosirius were evaluated using a polarized Nikon Eclipse 90i light microscope.

– For reticular fibers, tissues were stained with the Gomori's reticulin metal reduction method using 1% potassium permanganate for 1 min, followed by 2% sodium metabisulphite solution and sensibilization with 2% iron alum for 2 min. After that, samples were incubated in ammoniacal silver for 10–15 min and in 20% formaldehyde for 3 min. Finally, differentiation was performed with 2% gold chloride for 5 min and 2% thiosulphate for 1 min. No counterstaining agent was used.

– To evaluate elastic fibers, the orcein method was used. All samples were incubated in the orcein solution for 30 min at 37°and differentiated in acid-alcohol for a few seconds. No counterstaining agent was used.

4. To analyze the non-fibrillar components of the ECM, samples were stained as follows [14]:

– To determine the glycoproteins content in each tissue type, we used the Schiff Periodic acid staining method (PAS). Briefly, 0.5% periodic acid solution was used for 5 min as oxidant, followed by incubation in Schiff reagent for 15 min. Samples were slightly counterstained with Harris's hematoxylin for 20 seg.

– For analysis of proteoglycans, each tissue section was incubated in alcian blue solution for 30 min and then counterstained with nuclear fast red solution for 1 min.

### Immunohistochemistry

Detection of specific non-fibrillar components of the ECM -decorin, versican, agreccan and laminin- was carried out by immunohistochemistry. For antigen retrieval, deparaffinized tissue sections were incubated in pH 6 citrate buffer for 40 minutes at 95°C -laminin- or incubated with condroitinase ABC (Sigma-Aldrich) at 37°C for 1 h -decorin, versican and aggrecan-. Then, unspecific antigens were blocked with horse serum (Vector, Burlingame, CA, USA) and samples were incubated with primary antibodies anti-decorin (R&D systems, Minneapolis, MN), anti-versican (ABCam, Cambridge, UK) and anti-aggrecan (ABCam) or anti-laminin (Sigma-Aldrich, Steinheim, Germany) at a dilution of 1∶500, 1∶100, 1∶250, and 1∶1000, respectively, for 60 min at room temperature, except for laminin, which was incubated overnight at 4°C. Secondary antibodies were applied and the reaction was developed using a commercial 3-3′ diaminobenzidine kit (Vector Laboratories). Finally, samples were counterstained in Mayer's hematoxylin and mounted on coverslips for light microscopy evaluation. Expression of anti-smooth muscle actin (SMA) was identified by using pre-diluted anti-SMA primary antibodies (Master Diagnostica, Granada, Spain) for 30 min at room temperature and a secondary FITC-labeled antibody, and mounted with fluorescent DAPI-Vectashield (Vector Laboratories). To analyze cell proliferation, immunohistochemical analysis of PCNA was used using monoclonal anti-proliferating cell nuclear antigen clone PC10 (Sigma-Aldrich). First, cells were cultured in culture chambers and primary anti-PCNA antibodies were applied at a dilution of 1∶1000 for 60 min at room temperature. Then, secondary FITC-labeled antibodies were used for 30 min and samples were mounted using fluorescent DAPI-Vectashield.

Histological images were obtained at 200X magnification by using a Nikon Eclipse 90i light microscope, and the intensity of the staining signal was quantified for each specific ECM component by using ImageJ software as previously reported [15]. All images were taken and analyzed using exactly the same conditions (exposition time, white balance, background, etc.) for each tissue type.

### Gene expression analysis by microarray

Total mRNA was extracted and purified from each tissue -DDC, NPF and NFF- by using Qiagen RNeasy Mini Kit system (Qiagen, Mississauga, Ontario, Canada) following the manufacturer's instructions. Total RNA was converted into cDNA using a reverse transcriptase (Superscript II, Life Technologies, Inc., Carlsbad, California, EEUU) and a T7-oligo (dT) primer. Then, biotinilated cRNA was generated by using a T7 RNA polymerase and biotin-11-uridine-5′-triphosphate (Enzo Diagnostics, Farmingdale, Nueva York, EEUU). Labeled cRNA were chemically fragmented to facilitate the process of hybridization and hybridized to Affymetrix Human Genome U133 plus 2.0 oligonucleotide arrays for 6 hours at 45C. For the analysis of expression of ECM-related genes, we first selected all probe-sets with a role in the synthesis of ECM fibrillar components, glycosaminoglycans (GAG), proteoglycans and glycoproteins by using the information provided by Affymetrix. We also selected 6 WNT-pathway genes previously reported to be associated with DD [7]. If more than one probe-set was present in the array for the same gene, average expression values were obtained for that specific gene. To classify the three types of samples -DDC, NPF and NFF- according to their global gene expression profile, we performed hierarchical cluster analysis using the TM4 Software with all genes in the array [16]. All expression data are publically available at the public functional genomics data repository supporting MIAME-compliant data submissions Gene Expression Omnibus (http://www.ncbi.nlm.nih.gov/geo/query/acc.cgi?acc=GSE59746).

### Recultivation and cell proliferation analyses

Each tissue type was enzymatically digested in a 2 mg/ml *Clostridium hystoliticum* collagenase solution (Gibco BRL Life Technologies Ref. 17100-017, Karlsruhe, Germany) at 37C for 6 h. Isolated cells were harvested by centrifugation and cultured on tissue culture flasks using a Dulbecco's modified Eagle's medium (DMEM) supplemented with 10% fetal bovine serum (FBS) and 1% antibiotics. 10,000 cells of each tissue type were plated in 25 cm^2^ culture flasks, cultured in a 5% carbon dioxide atmosphere for 21 days, and the number of cells grown per mm^2^ of culture surface was quantified after 7, 14 and 21 days of culture in each tissue type. Culture medium was changed every three days, and cells were not trypsinized during the 21 days.

### Statistical analysis

For the global comparisons among the three tissue types -DDC, NPF and NFF-, we used the Kruskal-Wallis statistical test. To identify differences between two specific tissue types -DDC vs. NPF, DDC vs. NFF and NPF vs. NFF-, we used the Mann-Whitney test. All these tests were used to compare the signal intensity for the histochemical and immunohistochemical analyses (picrosirius, Gomori's reticulin, orcein, PAS, laminin, alcian blue, aggrecan, decorin and versican), the number of cells present in each tissue type and the number of cells showing positive expression of SMA. The analysis of gene expression levels as determined by microarray was carried out by using the U-rank statistical test as previously described [17]. This test allows detection of genes whose expression was higher for each of the samples corresponding to a specific group as compared to all samples in the other group. P values below 0.05 were considered statistically significant for all double-tailed tests.

## Results

### 1. Structural analysis of DDC, NPF and NFF human samples as determined by Masson's trichrome staining

**Figure 1:**
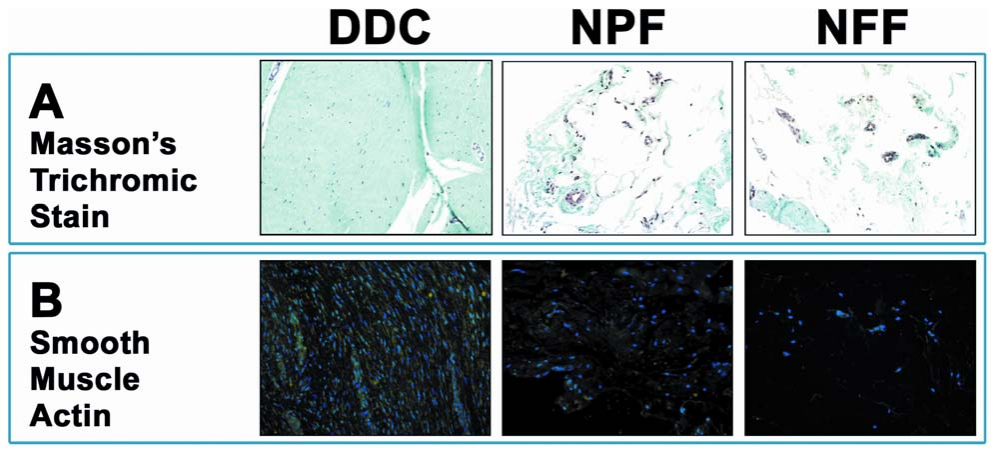
Histological analysis of Dupuytren's diseased contracture cords (DDC), palmar fascia clinically unaffected by Dupuytren's disease contracture (NPF), and normal forehand fascia (NFF). 1A: Analysis of tissue structure using Masson's trichrome staining. 1B: Analysis of expression of smooth muscle actin (SMA) by immunohistochemistry. Cell nuclei are stained in blue with DAPI and cells showing positive expression of SMA are labeled in green.

The analysis of human samples affected by Dupuytren's diseases (DDC) using Masson's trichrome staining revealed the presence of abundant fibrosis, with a fiber-rich dense tissue containing cells. In contrast, NFF normal tissues were characterized by few fibers and cells, with abundant blood vessels. Finally, NPF samples corresponding to hand palmar fascia tissue non-affected by Dupuytren's disease were very similar to NFF, with a slight increase of fibrous tissue (Figure 1A).

### 2. Analysis of cell density in DDC, NPF and NFF human samples

As shown in (Table 1 and Figure 1B), quantification of the number of cells per area of tissue demonstrated that DDC samples had significantly higher number of cells as compared with NPF and NFF (p<0.001). However, differences in the number of cells between NPF and NFF were not statistically significant (p>0.05).

**Table 1:** Quantification of the cell number and contents of key ECM components in each tissue type.

	Cell number	SMA	Picrosirius	Reticulin	Orcein	PAS	Laminin	Alcian blue	Aggrecan	Decorin	Versican
**DDC**	819.3±78.2	88.6±1.9	81.2±2.5	49.5±2.5	43.5±3.4	18.6±1.1	71.0±4.6	15.4±1.0	18.3±1.1	99.3±4.2	41.1±6.2
**NPF**	98.0±25.6	60.6±11.2	49.4±4.9	38.2±3.6	43.6±4.4	17.8±3.2	33.7±5.8	0.1±0.8	22.0±0.8	85.0±2.2	62.5±3.3
**NFF**	35.8±10.3	11.5±7.2	25.6±3.7	21.0±3.6	60.0±4.0	17.1±2.0	28.9±3.3	0.1±1.4	20.4±1.2	88.8±1.9	81.7±3.8
**NFP vs. NFF vs. DDC (KW)**	0.06081	0.00243*	0.00000*	0.00000*	0.00243*	0.11300	0.00000*	0.00000*	0.19080	0.00502*	0.00032*
**NPF vs. NFF (MW)**	0.51269	0.02000*	0.00036*	0.00027*	0.00705*	0.31708	0.79846	0.09373	0.75256	0.27986	0.04787*
**NPF vs. DDC (MW)**	0.04953*	0.04953*	0.00000*	0.02413*	0.88198	0.12577	0.00000*	0.00000*	0.27986	0.01854*	0.00002*
**DDC vs. NFF (MW)**	0.04953*	0.00101*	0.00000*	0.00000*	0.00101*	0.43422	0.00000*	0.00008*	0.15190	0.00150*	0.00072*

NPF: palmar fascia non affected by Dupuytren's disease contracture; DDC: Dupuytren's disease contracture cords; NFF: normal forehand fascia. Values correspond to average ± standard error. Cell number: quantification of the number of cells per area of tissue; SMA: smooth muscle actin. For each variable, the statistical p value for the global comparison using the Kruskal-Wallis test and for the one-to-one comparisons using the Mann-Whitney test are shown.

The analysis of expression of smooth muscle actin revealed that the percentage of cells with positive expression of this protein was significantly higher in DDC than in NPF and NFF, with NPF showing higher percentage of cells with positive expression of actin than NFF (p = 0.020) (Table 1 and Figure 1B).

### 3. Analysis of ECM fibrillar components in DDC, NPF and NFF human samples

**Figure 2:**
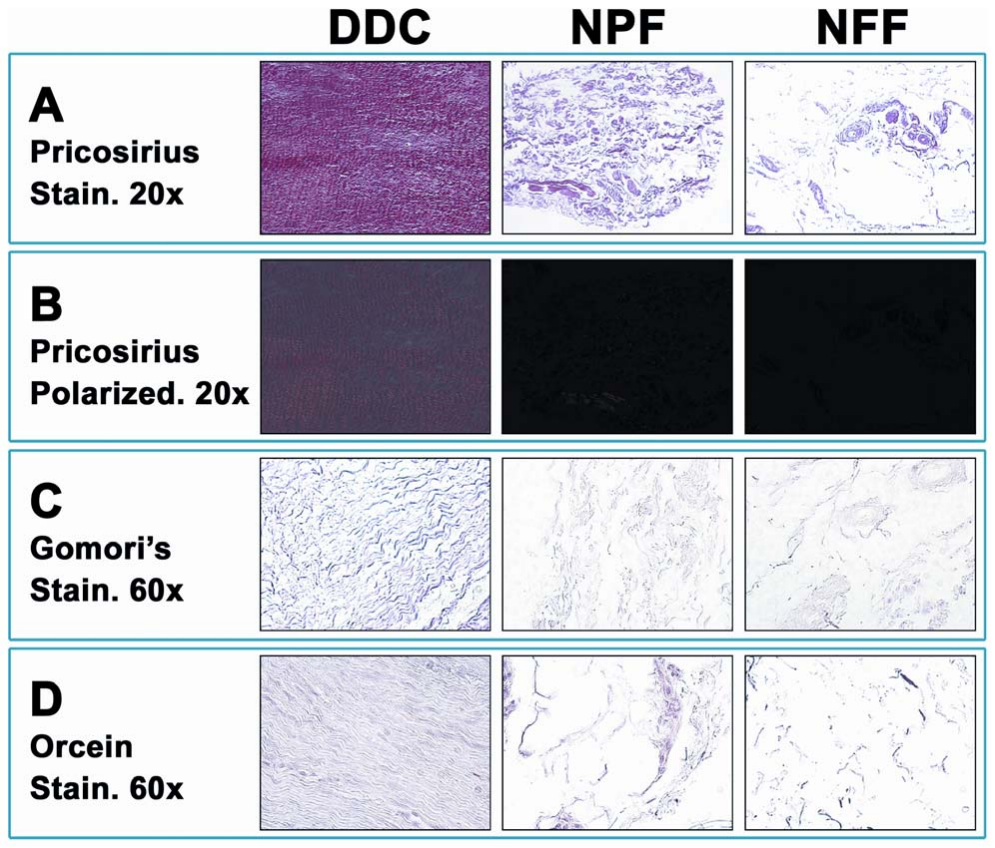
Analysis of the extracellular matrix fibrillar components of Dupuytren's diseased contracture cords (DDC), palmar fascia clinically unaffected by Dupuytren's disease contracture (NPF), and normal forehand fascia (NFF). 2A: Identification of collagen fibers as determined by picrosirius staining. 2B: Analysis of orientation of collagen fibers as determined by picrosirius staining using polarized microscopy. 2C: Staining of reticular fibers by using the technique of Gomori. 2D: Analysis of elastic fibers as determined by orcein staining.

Quantification of collagen fibers by picrosirius staining demonstrated that this fibrillar ECM component was significantly different among the three groups of samples analyzed in this work (p<0.001 for the Kruskal-Wallis test), with the highest collagen contents corresponding to DDC (81.2±2.5) and the lowest values corresponding to NFF (25.6±3.7) (Table 1 and Figure 2A). Differences were statistically significant for the comparison of DDC vs. NPF, DDC vs. NFF and NPF vs. NFF (p<0.01 for all three comparisons for the Mann-Whitney test). Interestingly, the analysis of collagen fibers using polarized light microscopy revealed that the abundant collagen mesh found in DDC was very organized and most fibers were oriented in the same direction. However, collagen fibers were oriented in different directions in NPF and NFF (Figure 2B). When all genes encoding for 46 collagen types were quantified at the mRNA level by microarray analysis (Table 2), we found that the expression of 14 (30.4%) collagen types was significantly different in NPF samples than in control NFF tissues (p<0.05 for the rank test). Of these 14 ECM components, 5 (35.7%) were downregulated in NPF (including some types of collagens 4, 8, 11, 14 and 22) and 9 (64.3%) were upregulated in NPF, including some types of collagens 4, 7, 8, 23, 24, 27, 28 and two procollagen isoforms. When NPF was compared to diseased DDC tissues, we found 11 collagen types differentially expressed between both tissue types, with 8 (72.7%) collagen types downregulated in NPF and 3 (27.3%) upregulated in DDC. Finally, the comparison of DDC with control NFF tissues found 17 types of collagen differentially expressed between both tissue types, with 14 (82.4%) of them overexpressed in DDC. The ratio of type III to type I collagen was 1.0676 in DDC, 1.0776 in NPF and 0.9956 in NFF.

**Table 2:** Microarray expression of ECM components and relevant WNT-pathway genes in the three tissue types analyzed in this work.

ECM COMPONENT OR PATHWAY	GENE SYMBOL	GENE TITLE	NPF	DDC	NFF
FIBERS	COL1A1	collagen, type I, alpha 1	3573.8	3686.9	3379.6
FIBERS	COL1A2	collagen, type I, alpha 2	5853	6139.6	5717.6
FIBERS	COL2A1	collagen, type II, alpha 1	8.1	6.5	4.6
FIBERS	COL3A1	collagen, type III, alpha 1	5079.1	5245.4	4528.5
FIBERS	COL4A1	collagen, type IV, alpha 1	268.5	230.1	287.3
FIBERS	COL4A2	collagen, type IV, alpha 2	259.9	246.6	261.2
FIBERS	COL4A3	collagen, type IV, alpha 3	4.4	5.8	6*
FIBERS	COL4A3BP	collagen, type IV, alpha 3 binding protein	184.4	218.3	192.4
FIBERS	COL4A4	collagen, type IV, alpha 4	22.4	40*	3.7*
FIBERS	COL4A5	collagen, type IV, alpha 5	17	12.3	18.5
FIBERS	COL4A6	collagen, type IV, alpha 6	3.6*	5.3	4.5
FIBERS	COL5A1	collagen, type V, alpha 1	1160.1	1087.2	1110.3
FIBERS	COL5A2	collagen, type V, alpha 2	1468.1	1617.4*	1235.4
FIBERS	COL5A3	collagen, type V, alpha 3	86.9	72.4	117.6
FIBERS	COL6A1	collagen, type VI, alpha 1	916.1	1074.2	922.5
FIBERS	COL6A2	collagen, type VI, alpha 2	1717.6*	2355.7*	1628.9
FIBERS	COL6A3	collagen, type VI, alpha 3	6846.1	7249.2	6261.3
FIBERS	COL6A6	collagen type VI alpha 6	50.9*	31.7	57.6
FIBERS	COL7A1	collagen, type VII, alpha 1	31.9*	49.9*	22.5*
FIBERS	COL8A1	collagen, type VIII, alpha 1	129.8	237.4	40.3*
FIBERS	COL8A2	collagen, type VIII, alpha 2	59.6	110.8	198.2*
FIBERS	COL9A1	collagen, type IX, alpha 1	1.3	1.7	1.3
FIBERS	COL9A2	collagen, type IX, alpha 2	13*	10.3	12.1
FIBERS	COL9A3	collagen, type IX, alpha 3	1.6	1.4	2
FIBERS	COL10A1	collagen, type X, alpha 1	27.4*	119.1*	9.5
FIBERS	COL11A1	collagen, type XI, alpha 1	91.1	143.4	82.8
FIBERS	COL11A2	collagen, type XI, alpha 2	25.2	23.8	29.5*
FIBERS	COL12A1	collagen, type XII, alpha 1	1016.6	1374.8*	1167.4
FIBERS	COL13A1	collagen, type XIII, alpha 1	93.2	119.7	68.5
FIBERS	COL14A1	collagen, type XIV, alpha 1	92.6*	35.6*	274*
FIBERS	COL15A1	collagen, type XV, alpha 1	108.8	322*	119.6
FIBERS	COL16A1	collagen, type XVI, alpha 1	845.6	956	755
FIBERS	COL17A1	collagen, type XVII, alpha 1	1.1*	1.8*	1.3
FIBERS	COL18A1	collagen, type XVIII, alpha 1	100.3	112.7	117.9
FIBERS	COL19A1	collagen, type XIX, alpha 1	3.5	1.4*	3.5
FIBERS	COL20A1	collagen, type XX, alpha 1	5.6	6.6	4
FIBERS	COL21A1	collagen, type XXI, alpha 1	65.7	63.2	94.6
FIBERS	COL22A1	collagen, type XXII, alpha 1	1.4	1.2*	5.1*
FIBERS	COL23A1	collagen, type XXIII, alpha 1	9.7	10.3*	3.3*
FIBERS	COL24A1	collagen, type XXIV, alpha 1	8.1	10.2*	3.4*
FIBERS	COL25A1	collagen, type XXV, alpha 1	1.8	2.3	2.6
FIBERS	COL27A1	collagen, type XXVII, alpha 1	36.5*	44.2*	22.8*
FIBERS	COL28A1	collagen, type XXVIII, alpha 1	5	4.4*	3*
FIBERS	COL29A1	collagen, type XXIX, alpha 1	3.5	4.8	2.6
FIBERS	PLOD1	procollagen-lysine, 2-oxoglutarate 5-dioxygenase 3	631.3*	721.6*	532.6*
FIBERS	PLOD2	procollagen-lysine, 2-oxoglutarate 5-dioxygenase 2	1314.9*	2072.8*	627.2*
FIBERS	ELN	elastin	748.9	552.1	725.4
FIBERS	FBN1	fibrillin 1	2461.8	1933.8*	3127.7*
FIBERS	FBN2	fibrillin 2	3.7*	8.5	10*
GAG	CHPF	chondroitin polymerizing factor	96.7	99*	135.9*
GAG	CHST1	carbohydrate (keratan sulfate Gal-6) sulfotransferase 1	16.3	9.8	13.4
GAG	CHST11	carbohydrate (chondroitin 4) sulfotransferase 11	47.1*	34.3	36.7
GAG	CHST12	carbohydrate (chondroitin 4) sulfotransferase 12	155.6*	195.1	183.4
GAG	CHST13	carbohydrate (chondroitin 4) sulfotransferase 13	3	1.8*	5.2*
GAG	CHST3	carbohydrate (chondroitin 6) sulfotransferase 3	82.8*	62.6*	83.1
GAG	CHSY1	chondroitin sulfate synthase 1	734.2	821.3*	582.8*
GAG	CHSY3	chondroitin sulfate synthase 3	90.1	102.1	76.2
GAG	CSGALNACT1	chondroitin sulfate N-acetylgalactosaminyltransferase 1	63.2*	133.8*	77.4
GAG	CSGALNACT2	chondroitin sulfate N-acetylgalactosaminyltransferase 2	466.3	506.6*	338.6*
GAG	CSGLCA-T	chondroitin sulfate glucuronyltransferase	224.2	246.8*	195.4*
GAG	CSPG4	chondroitin sulfate proteoglycan 4	43.1	34.6*	69.4*
GAG	CSPG4LYP1 & 2	chondroitin sulfate proteoglycan 4-like, Y-linked pseudogenes 1 & 2	2	1.7	1.7
GAG	CSPG5	chondroitin sulfate proteoglycan 5 (neuroglycan C)	4.7	4.7	5.8
GAG	DSE	dermatan sulfate epimerase	311.3*	509.1*	217*
GAG	DSEL	dermatan sulfate epimerase-like	319.2	263.5*	181.2*
GAG	HAS1	hyaluronan synthase 1	32.7	18.8	15
GAG	HAS2	hyaluronan synthase 2	623.1	832.3*	87.3*
GAG	HAS3	hyaluronan synthase 3	16.4	15.9*	10.1*
GAG	HGSNAT	heparan-alpha-glucosaminide N-acetyltransferase	115.1*	103.7*	133.4*
GAG	HS2ST1	heparan sulfate 2-O-sulfotransferase 1	155.1	134.9	173.4
GAG	HS3ST1	Heparan sulfate 3-O-sulfotransferase-1 precursor (3OST1)	2.2	2.6*	6.8*
GAG	HS3ST2	heparan sulfate (glucosamine) 3-O-sulfotransferase 2	70.1*	38	55.7
GAG	HS3ST3A1	heparan sulfate (glucosamine) 3-O-sulfotransferase 3A1	100.5	174.6*	83.6
GAG	HS3ST3B1	heparan sulfate (glucosamine) 3-O-sulfotransferase 3B1	180	227.6*	115*
GAG	HS3ST4	heparan sulfate (glucosamine) 3-O-sulfotransferase 4	8.9	8.4	7.9
GAG	HS3ST5	heparan sulfate (glucosamine) 3-O-sulfotransferase 5	23.6*	15.3*	23.8
GAG	HS3ST6	heparan sulfate (glucosamine) 3-O-sulfotransferase 6	3.7*	9.1	7.7
GAG	HS6ST1	heparan sulfate 6-O-sulfotransferase 1	55.1	68.6	63.5
GAG	HS6ST2	heparan sulfate 6-O-sulfotransferase 2	3.2*	1.1*	4.8
GAG	HS6ST3	heparan sulfate 6-O-sulfotransferase 3	4.4	3.9	5.3
GAG	NDST1	N-deacetylase/N-sulfotransferase (heparanglucosaminyl) 1	82.2	88.9	91.7
GAG	NDST2	N-deacetylase/N-sulfotransferase (heparanglucosaminyl) 2	71.8*	64.7*	79.7*
GAG	NDST3	N-deacetylase/N-sulfotransferase (heparanglucosaminyl) 3	15.2*	5.6*	3.1*
GAG	NDST4	N-deacetylase/N-sulfotransferase (heparanglucosaminyl) 4	5.9*	0.8*	3.6
GLYCOPROTEINS	FN1	fibronectin 1	4547.3*	4770.6	4229.1
GLYCOPROTEINS	LAMA1	laminin, alpha 1	73.1	83.5	67.5
GLYCOPROTEINS	LAMA2	laminin, alpha 2	187.3	184	207.2
GLYCOPROTEINS	LAMA3	laminin, alpha 3	12.7	13.1*	10*
GLYCOPROTEINS	LAMA4	laminin, alpha 4	186	198.6	181.1
GLYCOPROTEINS	LAMA5	KIAA0533 protein	26.9*	15*	17.4*
GLYCOPROTEINS	LAMB1	laminin, beta 1	734.2	847.8	872.5
GLYCOPROTEINS	LAMB2	laminin, beta 2 (laminin S)	255.4*	278.7*	282.4*
GLYCOPROTEINS	LAMB2L	laminin, beta 2-like	8.7*	12.4	13.8*
GLYCOPROTEINS	LAMB3	laminin, beta 3	33.4	48.8	30.7
GLYCOPROTEINS	LAMB4	laminin, beta 4	2*	4.3*	6.9*
GLYCOPROTEINS	LAMC1	laminin, gamma 1 (formerly LAMB2)	1942	1534.3	2074.2
GLYCOPROTEINS	LAMC2	laminin, gamma 2	21	21.6	77
GLYCOPROTEINS	LAMC3	laminin, gamma 3	5.3	7.2	7.4*
GLYCOPROTEINS	NID1	nidogen 1_ENTACTIN	227.1	249.1*	443.7*
GLYCOPROTEINS	NID2	nidogen 2 (osteonidogen)	501.8	477	489
GLYCOPROTEINS	SPARC	secreted protein, acidic, cysteine-rich (osteonectin)	4443.1	4715.8	4462.4
GLYCOPROTEINS	TNC	tenascin	1129.3	1402.5	988.4
PROTEOGLYCANS	ACAN	aggrecan	70.3*	13.1*	93.3
PROTEOGLYCANS	BGN	biglycan	569.2*	752.1*	400.6*
PROTEOGLYCANS	DCN	decorin	6754.8*	7088.3	6774
PROTEOGLYCANS	HSPG2	heparan sulfate proteoglycan 2_PERLECAN	345.8	333	371
PROTEOGLYCANS	LUM	lumican	2894	3220.6	2536.3
PROTEOGLYCANS	NCAN	neurocan	9.2*	1.4*	12.3*
PROTEOGLYCANS	SDC1	syndecan 1	76.2	117.6	87
PROTEOGLYCANS	SDC2	syndecan 2	400.2	293.7*	753.9*
PROTEOGLYCANS	SDC3	syndecan 3	35.7	38.7	40.8
PROTEOGLYCANS	SDC4	syndecan 4	969	1075*	942
PROTEOGLYCANS	SDCBP	syndecan binding protein (syntenin)	3358.1	3840.6	3826.7*
PROTEOGLYCANS	SDCBP2	syndecan binding protein (syntenin) 2	30.1	33.3	33.5
PROTEOGLYCANS	VCAN	versican	1542.5	1141.5*	1815.9
WNT PATHWAY	RSPO2	R-spondin 2 homolog (Xenopus laevis)	9.3*	2.8*	17.3
WNT PATHWAY	SFRP4	secreted frizzled-related protein 4	3017.9	2516.3	717.7*
WNT PATHWAY	SULF1	sulfatase 1	502.8	450.5	319.9
WNT PATHWAY	WNT2	wingless-type MMTV integration site family member 2	14.7	15.5	21.7
WNT PATHWAY	WNT4	wingless-type MMTV integration site family, member 4	1.4*	3.6	3.2*
WNT PATHWAY	WNT7B	wingless-type MMTV integration site family, member 7B	5.8	8.5*	6.4

Each gene was classified as a fibrillar ECM component (fibers), glycosaminoglycan (GAG), glycoprotein or proteoglycan. NPF: palmar fascia non affected by Dupuytren's disease contracture; DDC: Dupuytren's disease contracture cords; NFF: normal forehand fascia. Statistically significant differences for the U-rank test are labeled with asterisks: in the NPF column, asterisks show statistically significant differences for the comparison of NPF vs. DDC samples; in the DDC column, for the comparison DDC vs. NFF; in the NFF column, for the NFF vs. NPF comparison.

The analysis of reticular fibers in DDC, NPF and NFF human samples (Table 1 and Figure 2C) showed that the amount of reticular fibers as determined by reticulin staining technique was significantly different among the three sample types (p<0.001 for the Kruskal-Wallis test). Specifically, the highest content in reticular fibers (49.5±2.5) was found in DDC tissues, which was significantly higher as compared to NPF (38.2±3.6; p = 0.0241 for the Mann-Whitney test) and NFF (21.0±3.6; p<0.001). At the RNA levels (Table 2), the highest expression values of the collagen 3 gene corresponded to DDC samples, which were very similar to those of NPF samples, whilst the lowest expression was found in NFF (differences were not significant).

On the other hand, identification of elastic fibers by orcein staining revealed that some differences exist among the three sample types (p = 0.002 for the Kruskal-Wallis test). As shown in Table 1 and Figure 2D, NFF samples had significantly higher content in elastic fibers (60.0±4.0) than NPF (43.6±4.4; p = 0.007 for the Mann-Whitney test) and DDC (43.5±3.4; p = 0.001). The same trend was found at the RNA level (Table 2), with the highest expression values of fibrillin 1 and 2 found in NFF, although the highest expression of the elastin gene was found in NPF followed by NFF tissues.

### 4. Analysis of ECM non-fibrillar components in DDC, NPF and NFF human samples

**Figure 3:**
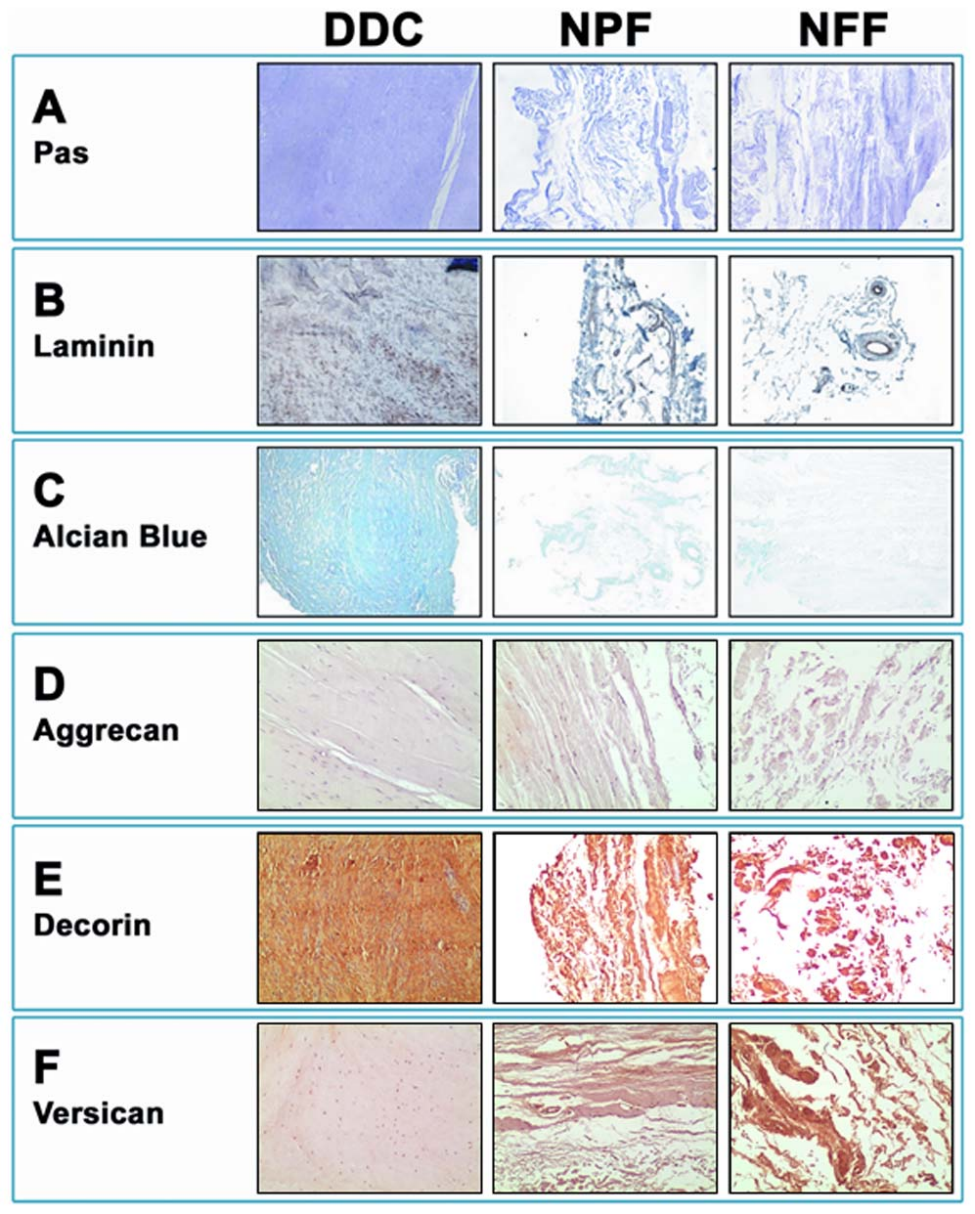
Histochemical and immunohistochemical analysis of Dupuytren's diseased contracture cords (DDC), palmar fascia clinically unaffected by Dupuytren's disease contracture (NPF), and normal forehand fascia (NFF). 3A: Detection of glycoproteins by PAS staining. 3B: Laminin staining by immunohistochemistry. 3C: Quantification of proteoglycans by alcian blue staining. 3D: Aggrecan staining by immunohistochemistry. 3E: Decorin staining by immunohistochemistry. 3F: Versican staining by immunohistochemistry.

First, the analysis of glycoproteins was carried out by using the periodic acid–Schiff (PAS) staining method. As shown in Table 1 and Figure 3A, differences among the three tissue types (DDC, NPF and NFF) were not statistically significant. However, quantification of the multiadhesive glycoprotein laminin by immunohistochemistry revealed the existence of significant differences for the global comparison of the three tissue samples (p<0.001 for the Kruskal-Wallis test). DDC specimens had significantly higher laminin content (71.0±4.6) than NPF (33.7±5.8; p<0.001) and NFF (28.9±3.3; p<0.001), although no differences existed between these two later samples (Table 1 and Figure 3B). The microarray analysis of genes encoding for 13 laminin types (Table 2) showed that the highest expression values of 4 laminin types were found in DDC tissues, whereas 8 laminins were overexpressed in NFF. The gene expression of 6 laminin types was statistically different between NPF and control NFF samples, with LAMC3, LAMB2, LAMB2L and LAMB4 genes downregulated in NPF. 4 laminin genes were statistically different between NPF and DDC, with only one component being higher in NPF (LAMA5), and 4 genes were significantly different between DDC and NFF. The expression levels of 5 other glycoproteins included in the array system -NID1, NID2, SPARC, FN1 and TNC- did not differ among the 3 samples, with the only exception of NID1 (entactin gene), which was significantly higher in NFF and FN1 (fibronectin 1), which was significantly higher in NFF than in DDC and NPF.

Then, quantification of ECM proteoglycans by alcian blue staining demonstrated that the amount of these components differed among the three sample types (p<0.001 for the Kruskal-Wallis test), with the highest values corresponding to DDC (15.4±1.0), which were significantly higher than those found in NPF (0.1±0.8; p<0.001) and NFF (0.1±1.4) (Table 1 and Figure 3C). The immunohistochemical analysis of specific proteoglycans showed that global significant differences existed among all sample types -DDC, NPF and NFF- for decorin and versican (p<0.001 for the Kruskal-Wallis test) (Table 1 and Figures 3D, 3E and 3F, respectively). In addition, decorin protein expression was significantly higher in DDC as compared to NPF and NFF, with no differences between these two tissue types, and the same trend was found at the mRNA level. However, versican showed the reverse behavior, with significantly higher expression in NFF tissues and the lowest expression corresponding to DDC at both the protein and the mRNA levels. Interestingly, aggrecan expression was very low in all samples types, with no significant differences among samples at the protein level, although its expression was significantly higher in NFF and lower in DDC at the mRNA level. At the mRNA level, the analysis of genes encoding for 13 proteoglycan ECM components showed downregulation of 4 (30.8%) of these genes in NPF as compared to NFF (neurocan, biglycan, syntenin and syndecan 2), with biglycan overexpressed in NFP. The same number of proteoglycans genes (4 genes, 30.8%) was differentially expressed between NPF and DDC, with 2 proteoglycans genes upregulated in NPF (aggrecan and neurocan) and 2 upregulated in DDC (decorin and biglycan). The comparison of DDC samples vs. NFF tissues demonstrated that 4 components -VCAN, ACAN, SDC2 and NCAN- were upregulated in NFF and 2 components were overexpressed in DDC -SDC4 and BGN-.

Finally, quantification of genes with a role in glycosaminoglycan synthesis by microarray analysis -35 GAG components- (Table 2) revealed that 15 -42.9%- of these components were differentially expressed between NPF and NFF samples (NDST3, CHSY1, CSGALNACT2, CSGLCA-T, DSEL, HAS2, HAS3, HS3ST3B1 and DSE were overexpressed in NPF and HGSNAT, NDST2, CHPF, CHST13, CSPG4 and HS3ST1 in NFF); 13–37.1%- GAG components were significantly different between NPF and DDC, with 4 components overexpressed in DDC and 9 in NPF; and 21–60%- GAG types were significantly different between DDC and NFF, with 11 overexpressed in DDC and 10 in NFF.

### 5. Analysis of key WNT-pathway genes in DDC, NPF and NFF human samples

The analysis of six WNT genes previously reported to be disregulated in DD revealed that the expression of these genes was very low in all sample types, except for SFRP4 and SULF1. As shown in Table 2, the highest expression of both genes was found in DDC and NPF, and the lowest expression corresponded to NFF.

### 6. Unsupervised cluster analysis of DDC, NPF and NFF human samples

**Figure 4:**
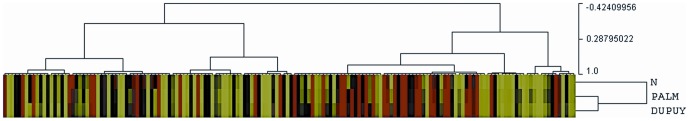
Unsupervised hierarchical cluster analysis of the different samples included in the present study. Overexpressed genes are shown in green and downregulated genes are shown in red. The classification tree of the samples is displayed at the right side of the figure. N: normal forehand fascia (NFF); Palm: palmar fascia clinically unaffected by Dupuytren's disease contracture (NPF); Dupuy: Dupuytren's diseased contracture cords (DDC).

When all genes/EST included in the microarray system were used to classify all samples by unsupervised cluster analysis, we found that DDC samples tended to cluster together with NPF in one branch of the hierarchical classification tree, whereas NFF samples clustered in the other branch (Figure 4).

### 7. Cell proliferation analysis of cell cultures of DDC, NPF and NFF human samples

**Figure 5:**
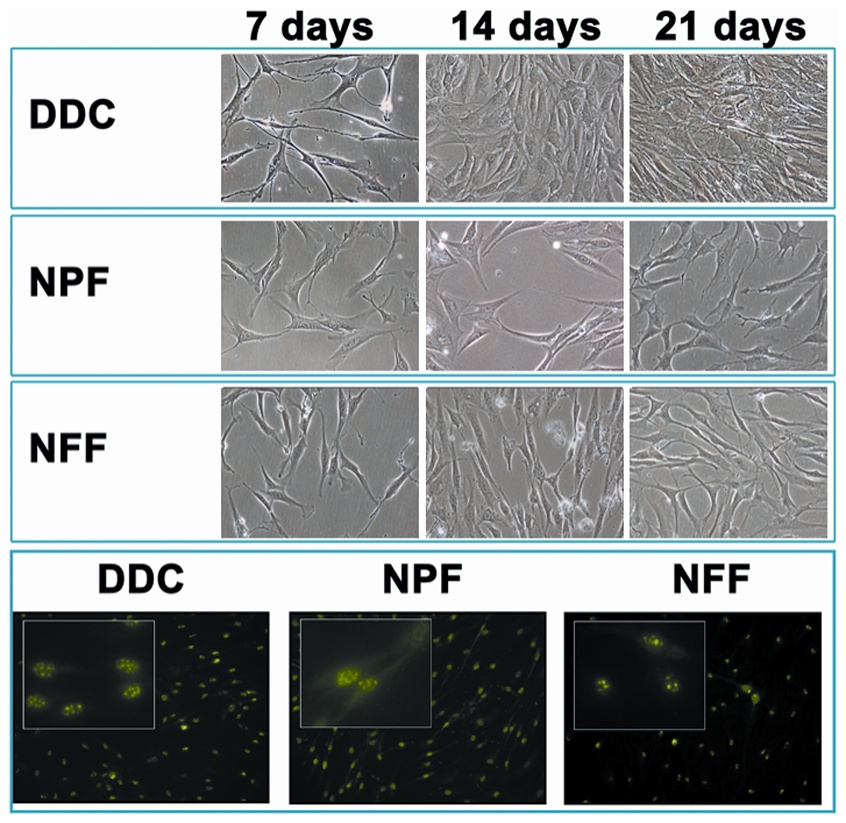
Recultivation and cell proliferation analyses of Dupuytren's diseased contracture cords (DDC), palmar fascia clinically unaffected by Dupuytren's disease contracture (NPF), and normal forehand fascia (NFF). The top panel shows representative images of cells cultured from each tissue type after 7, 14 and 21 days of culture, and the lowest panel shows the analysis of cell proliferation using PCNA immunohistochemistry with two magnification levels.

When the three types of samples -DDC, NPF and NFF- were subjected to enzymatic digestion and released cells were cultured ex vivo, we found that cells isolated from DDC tended to proliferate faster (average 22, 75 and 125 cells per mm^2^ after 7, 14 and 21 days, respectively) than cells isolated from NPF (average 20, 49 and 65 cells per mm^2^ after 7, 14 and 21 days, respectively) and NFF (average 18, 35 and 43 cells per mm^2^ after 7, 14 and 21 days, respectively) (Figure 5), with higher number of cells in the DDC group than in the NPF and NFF groups after 21 days of culture (p = 0.029). Differences between NPF and NFF were also significant (p>0.029). Strikingly, all cultured cells were positive for the cell-proliferation marker PCNA.

## Discussion

Numerous previous works already demonstrated that Dupuytren's disease is a complex condition in which a large variety of genes are involved [2], [18], [19], [20]. However, this is one of the first studies focused on the evaluation of the palmar fascia that is not clinically affected by the fibrous cord of this disease but is anatomically related to this tissue (NPF tissues), and normal NFF tissues using a comprehensive approach.

According to our results, the global gene expression profile of NPF samples was similar to that of DDC tissues and differed from the expression showed by normal NFF samples. This finding implies that NPF cells could share important similarities with DDC cells, suggesting that NPF tissues could not be normal from a gene expression standpoint even though these are apparently clinically unaffected. To shed light on this issue, we first quantified the number of cells present in each tissue and determined the percentage of cells that were positive for smooth muscle actin, a marker of myofibroblasts. Several reports [3], [12] previously demonstrated that contraction of the palmar and digital cords may be induced by a 4- to 20- fold increase in the cells of these structures [21], and a transformation of normal palmar fibroblasts into myofibroblasts during the first phases of the Dupuytren's disease. During the proliferative phase of the disease, it is thought that uncontrolled proliferation of myofibroblasts leads to the formation of nodules, resembling fibroma [22]. In this regard, our results showed that the number of cells per area of diseased tissue (DDC) was very high and cells expressed high amounts of smooth muscle actin, thus confirming the abundance of myofibroblasts in Dupuytren's diseased tissue. These values were statistically higher in DDC than NPF and NFF. Interestingly, both the cell number and the percentage of actin-positive cells showed different values in NPF tissues -which are typically considered as normal tissues non-affected by the disease- and in control NFF, with higher values in NPF than in NFF, although differences were not statistically significant for the number of cells. In consequence, we could hypothesize that palmar NPF tissues may also be affected by the disease, although at lower extent than Dupuytren's disease contracture cords. Since myofibroblasts could act as mediators for the disease generation and progression, leading to progressive flexion deformity of the involved fingers [23], [24], the identification of a high amount of these cells in an area of the palm fascia traditionally considered as healthy tissue could be clinically relevant.

Strikingly, our *ex vivo* cell culture assays found that cells corresponding to NPF were able to proliferate in culture at a significantly higher rate than normal FFN cells, although at lower rate than diseased DDC cells, but the expression of the proliferation marker PCNA was similar. These results are in agreement with our idea that NPF cells could be pathological, although at lower extent than diseased cells of the fibrous cord, at least at this stage. Previous reports already demonstrated that cells cultured from tissues affected by Dupuytren's disease may have higher proliferation rate than control tissues [25], [26], [27], and several authors found a significant overexpression of several genes involved in cell proliferation in these cells [28]. From a translational standpoint, these results suggest that tissues that are apparently unaffected by DD such as NPF could be also affected by the disease. These results are in agreement with previous reports suggesting that clinically-unaffected palmar skin of DD patients may have important alterations related to the disease [29].

Once the cells of each tissue type were characterized both in situ and in culture, we carried out a study of the ECM of these tissues by immunohistochemistry, histochemistry and microarray. This study confirmed that DDC tissues had increased extracellular matrix (ECM) deposition as compared to NPF and NFF, as previously suggested by Rehman and cols [30] and by Ratkaj and cols [28]. In this sense, one of the most important ECM components is the fibrillar component, which typically becomes very abundant in Dupuytren's disease [11], [21], [31], and a major biochemical abnormality found in Dupuytren's tissue is an increase in total collagen associated with an increase in the ratio of type III to type I collagen [21]. In this regard, our results demonstrated that DDC had significantly more collagen content than NPF and NFF as determined by picrosirius and Masson's trichrome staining, and that collagen fibers were highly organized and oriented only in DDC tissues, with an increase in the ratio of type III to type I collagen as compared to controls. The concentration of collagen fibers oriented in the same direction is one of the main factors related to the pathogenicity of this disease, in which contracture cords are predominantly composed of an oriented fibrous structure [32] mainly consisting of collagen fibers [12]. Our histological analysis also revealed that the amount of collagen fibers in NPF almost duplicated the amount found in control NFF, with the ratio of type III to type I collagen being similar in DDC and NPF. These results again suggest that NPF tissues should not be considered as normal and specific medical and surgical procedures could be indicated in future protocols for treatment of this area of the hand palm. This is in agreement with the mRNA analysis as determined by microarray, which found that NPF tissues only differed from DDC tissues in 23.9% of collagen-related genes, but differed from NFF in 30.4% of these genes. It is well known that most collagen fibrils are comprised in vivo of several collagen types, including collagens I, II, III, V, IX, and XI [33]. In this regard, our results put forward that DDC tissues express the highest amounts of collagens types I, III and Vα2, suggesting that these diseased tissues could be constituted by a well-developed fibrillar mesh of heterogeneous collagen fibers. In addition, we found an alteration in the expression of other fibrillar collagens such as collagen XXVII, which forms thin non-striated fibrils [34], and the fibril-associated collagens XII and XV. All this could explain the biomechanical behavior of Dupuytren's disease cords. Moreover, collagens types V and XIV play a role in regulating fibrillogenesis by controlling the initiation of collagen fibril assembly [35], [36]. The significant alteration of both collagens that we found in DDC tissues points to the idea that the complex process of collagen fibrils formation is disregulated in Dupuytren's disease, as previously suggested [28], [30]. Beside this, our analysis of non-fibrillar collagens such as collagens types IV, VI, VII, VIII and X revealed a significant alteration of these collagens as well, suggesting that the diffuse tissue collagen network of normal connective tissues could also be altered in DDC and NPF.

In this work, we also quantified the presence of important ECM fibers that are very seldom analyzed in Dupuytren's disease. First, reticular fibers were significantly more abundant in DDC than in the other sample types, but NPF showed significantly more reticular fibers than control NFF, suggesting again that DDC and NPF could have increased biomechanical properties than control tissues. Then, the analysis of elastic fibers revealed a significant reduction of these fibers in both the DDC and the NPF tissues as compared to control NFF, with no differences between DDC and NPF. This could explain the lack of flexibility typically found in Dupuytren's disease contracture cords and supports again the idea that NPF tissues could not be normal. The results found for all fibrillar components of the ECM show a different fibrillar pattern among the three types of tissues. As a consequence, the biomechanical properties could be different in each group, with palmar tissues being stiffer and less elastic than control NFF.

On the other hand, non-fibrillar ECM components play key roles in cell-cell interaction, cell adhesion, proliferation migration and response, and they are essential for the maintenance of the 3D structure and hydration level of human tissues [14], [37]. Due to their crucial function, alteration of these ECM molecules may be associated to tissue dysfunction and pathology. The first type of non-fibrillar components that we analyzed in the present work are the proteoglycans. Most proteoglycans consist of a core protein with several glycosaminoglycan (GAG) chains attached [38], and these complex structures play a key role in regulating the transit of ECM molecules, including water, throughout the tissue matrix. Our histological analysis using PAS staining methods showed that the concentration of glycoproteins was very similar among the three tissue types analyzed in the present work, with a non-significant increment in DDC tissues. However, the analysis of specific proteoglycans revealed that some of these ECM components were indeed differentially expressed among the three tissue types. At the mRNA level, we found that the percentage of genes differentially expressed between NPF and NFF was the same found for the comparison of DDC vs. NFF (30.8%). This finding is in agreement with our hypothesis that both DDC and NPF tissues have important ECM alterations in vivo. To confirm this hypothesis, we analyzed three important individual proteoglycans in tissue samples corresponding to DDC, NPF and NFF by immunohistochemistry and microarray. The results of this analysis showed that both decorin and versican were significantly altered in DDC. Previous works suggest that both proteoglycans play important roles in the formation of interstitial collagen fibers by regulating collagen fibrillogenesis and the assembly of fibrils into fibers [38], [39], cell migration and adhesion [40] and fibroblast proliferation [41]. The alteration of these components in DDC and NPF tissues could be associated to the disregulation found for the fibrillar ECM components and suggests again that NPF tissues may not be histologically normal.

Related with proteoglycans, glycosaminoglycans (GAG) are important ECM components with a role in the synthesis, maintaining and physiology of the ECM. In this regard, the microarray analysis of gene transcripts corresponding to genes involved in the synthesis of several GAG showed that 42.9% of these genes were differentially expressed between NPF tissues and control NFF samples, suggesting again that NPF tissues may be not histologically normal. 37.1% of all GAG genes were differentially expressed between DDC and NPF, probably due to the fact that NPF tissues do not harbor the high level of damage of DDC tissues.Finally, glycoproteins are abundant in the ECM of most tissues, with higher concentration at the basement lamina, especially laminin. Laminin is a large family of heterodimeric proteins involved in the formation of networks and filaments working as cell bindings along with integrins and other components [37], [42]. The analysis of laminin in samples included in the present work revealed that the highest expression corresponded to DDC, with significantly lower levels in NPF and NFF. Previous studies reported that laminin could be upregulated in proliferative nodules of Dupuytren's disease, although it may be restricted to these nodules [43]. Several isoforms of laminin have been found altered in many tissues, including human tumors, and overexpression of this glycoprotein could be associated to tumor progression, migration and invasion [44]. The increment of laminin protein in DDC could explain the increased cell proliferation found in these tissues. Interestingly, the laminin protein levels found in NPF were again higher than those of control NFF and lower than diseased DDC. Another important glycoprotein that we found overexpressed in DDC at the mRNA level is fibronectin. This increment is in agreement with previous works demonstrating that Dupuytren's disease nodules and fibrotic cords contained increased amounts of collagen, fibronectin and proteoglycans [45].

To confirm all these results, we also analyzed the expression of 6 relevant genes with a role in the WNT pathway as suggested by Dolmans and cols [7]. WNT genes are known to encode glycoproteins and extracellular signaling molecules, and this pathway has been found altered in cancer and DD [7]. In our study, we found that 4 of these genes were absent or expressed at very low levels, although the genes SFRP4 and SULF1 were highly expressed. It is remarkable that the lowest expression of both genes corresponded to normal NFF, whereas DDC and NPF samples had similarly high expression. Disregulation of the expression levels of both genes has been associated to an alteration of the synthesis of proteoglycans and beta-catenin degradation, which could trigger fibroblast proliferation in DD [7], [46].

In conclusion, this is one of the first studies in which the main components of the ECM matrix were studied and quantified not only in controls and tissues affected by Dupuytren's disease, but also in palmar fascia clinically unaffected by Dupuytren's disease contracture (NPF) using microarray approaches, histological, histochemical, immunohistochemical approaches and cell recultivation methods. Although previous works already demonstrated that palmar skin of DD patients may be affected by the disease in the absence of clinically detectable symptoms [29], the results of this comprehensive approach confirm that DDC tissues have intense ECM alterations, and demonstrate for the first time that NPF tissues should not be considered as normal tissues. The clinical and translational consequences of this could be important, since these results allow us to establish that different degrees of alteration could affect the whole palmar fascia, with areas clinically affected by DD -areas showing fibrotic cords- and areas affected by the disease without clinical manifestations. Therefore, unaffected palm regions should not be considered as normal. If our results are confirmed in larger series of cases, a modification of the therapeutic approach used for the treatment of Dupuytren's disease, including removal or drug treatment of the remaining palm fascia, should be considered.

## References

[1] Rehman S, Goodacre R, Day PJ, Bayat A, Westerhoff HV (2011) Dupuytren's: a systems biology disease. Arthritis Res Ther 13: 238. 21943049 10.1186/ar3438 PMC3308066

[2] Michou L, Lermusiaux JL, Teyssedou JP, Bardin T, Beaudreuil J, et al (2012) Genetics of Dupuytren's disease. Joint Bone Spine 79: 7–12. 21803632 10.1016/j.jbspin.2011.05.027

[3] Wilkinson JM, Davidson RK, Swingler TE, Jones ER, Corps AN, et al (2012) MMP-14 and MMP-2 are key metalloproteases in Dupuytren's disease fibroblast-mediated contraction. Biochim Biophys Acta 1822: 897–905. 22342364 10.1016/j.bbadis.2012.02.001

[4] Shih B, Bayat A (2010) Scientific understanding and clinical management of Dupuytren disease. Nat Rev Rheumatol 6: 715–726. 21060335 10.1038/nrrheum.2010.180

[5] Verhoekx JS, Verjee LS, Izadi D, Chan JK, Nicolaidou V, et al (2013) Isometric contraction of Dupuytren's myofibroblasts is inhibited by blocking intercellular junctions. J Invest Dermatol 133: 2664–2671. 23652794 10.1038/jid.2013.219

[6] Satish L, LaFramboise WA, O'Gorman DB, Johnson S, Janto B, et al (2008) Identification of differentially expressed genes in fibroblasts derived from patients with Dupuytren's Contracture. BMC Med Genomics 1: 10. 18433489 10.1186/1755-8794-1-10 PMC2377253

[7] Dolmans GH, Werker PM, Hennies HC, Furniss D, Festen EA, et al (2011) Wnt signaling and Dupuytren's disease. N Engl J Med 365: 307–317. 21732829 10.1056/NEJMoa1101029

[8] Thomas A, Bayat A (2010) The emerging role of Clostridium histolyticum collagenase in the treatment of Dupuytren disease. Ther Clin Risk Manag 6: 557–572. 21127696 10.2147/TCRM.S8591 PMC2988615

[9] Bainbridge C, Gerber RA, Szczypa PP, Smith T, Kushner H, et al (2012) Efficacy of collagenase in patients who did and did not have previous hand surgery for Dupuytren's contracture. J Plast Surg Hand Surg 46: 177–183. 22670890 10.3109/2000656X.2012.683795 PMC3469218

[10] Sampson S, Meng M, Schulte A, Trainor D, Montenegro R, et al (2011) Management of Dupuytren contracture with ultrasound-guided lidocaine injection and needle aponeurotomy coupled with osteopathic manipulative treatment. J Am Osteopath Assoc 111: 113–116. 21357497

[11] Kaplan FT (2011) Collagenase clostridium histolyticum injection for the treatment of Dupuytren's contracture. Drugs Today (Barc) 47: 653–667. 21971540 10.1358/dot.2011.47.9.1656502

[12] Black EM, Blazar PE (2011) Dupuytren disease: an evolving understanding of an age-old disease. J Am Acad Orthop Surg 19: 746–757. 22134207 10.5435/00124635-201112000-00005

[13] Rayan GM (1999) Palmar fascial complex anatomy and pathology in Dupuytren's disease. Hand Clin 15: 73–86, vi-vii. 10050244

[14] Oliveira AC, Garzon I, Ionescu AM, Carriel V, Cardona Jde L, et al (2013) Evaluation of small intestine grafts decellularization methods for corneal tissue engineering. PloS one 8: e66538. 23799114 10.1371/journal.pone.0066538 PMC3682956

[15] Carriel VS, Aneiros-Fernandez J, Arias-Santiago S, Garzon IJ, Alaminos M, et al (2011) A novel histochemical method for a simultaneous staining of melanin and collagen fibers. J Histochem Cytochem 59: 270–277. 21378281 10.1369/0022155410398001 PMC3201150

[16] Saeed AI, Sharov V, White J, Li J, Liang W, et al (2003) TM4: a free, open-source system for microarray data management and analysis. Biotechniques 34: 374–378. 12613259 10.2144/03342mt01

[17] Berdasco M, Alcazar R, Garcia-Ortiz MV, Ballestar E, Fernandez AF, et al (2008) Promoter DNA hypermethylation and gene repression in undifferentiated Arabidopsis cells. PloS one 3: e3306. 18827894 10.1371/journal.pone.0003306 PMC2556100

[18] Ojwang JO, Adrianto I, Gray-McGuire C, Nath SK, Sun C, et al (2010) Genome-wide association scan of Dupuytren's disease. J Hand Surg Am 35: 2039–2045. 20971583 10.1016/j.jhsa.2010.08.008 PMC2998563

[19] Satish L, Gallo PH, Baratz ME, Johnson S, Kathju S (2011) Reversal of TGF-beta1 stimulation of alpha-smooth muscle actin and extracellular matrix components by cyclic AMP in Dupuytren's-derived fibroblasts. BMC Musculoskelet Disord 12: 113. 21612641 10.1186/1471-2474-12-113 PMC3125251

[20] Satish L, LaFramboise WA, Johnson S, Vi L, Njarlangattil A, et al (2012) Fibroblasts from phenotypically normal palmar fascia exhibit molecular profiles highly similar to fibroblasts from active disease in Dupuytren's Contracture. BMC Med Genomics 5: 15. 22559715 10.1186/1755-8794-5-15 PMC3375203

[21] Murrell GA, Francis MJ, Bromley L (1991) The collagen changes of Dupuytren's contracture. J Hand Surg Br 16: 263–266. 1960490 10.1016/0266-7681(91)90050-x

[22] Picardo NE, Khan WS (2012) Advances in the understanding of the aetiology of Dupuytren's disease. Surgeon 10: 151–158. 22297148 10.1016/j.surge.2012.01.004

[23] Vi L, Njarlangattil A, Wu Y, Gan BS, O'Gorman DB (2009) Type-1 Collagen differentially alters beta-catenin accumulation in primary Dupuytren's Disease cord and adjacent palmar fascia cells. BMC Musculoskelet Disord 10: 72. 19545383 10.1186/1471-2474-10-72 PMC2716298

[24] Vi L, Feng L, Zhu RD, Wu Y, Satish L, et al (2009) Periostin differentially induces proliferation, contraction and apoptosis of primary Dupuytren's disease and adjacent palmar fascia cells. Exp Cell Res 315: 3574–3586. 19619531 10.1016/j.yexcr.2009.07.015 PMC5017872

[25] Hindocha S, Iqbal SA, Farhatullah S, Paus R, Bayat A (2011) Characterization of stem cells in Dupuytren's disease. Br J Surg 98: 308–315. 21104823 10.1002/bjs.7307

[26] Iqbal SA, Manning C, Syed F, Kolluru V, Hayton M, et al (2012) Identification of mesenchymal stem cells in perinodular fat and skin in Dupuytren's disease: a potential source of myofibroblasts with implications for pathogenesis and therapy. Stem Cells Dev 21: 609–622. 21612554 10.1089/scd.2011.0140 PMC3280606

[27] Luck JV (1959) Dupuytren's contracture; a new concept of the pathogenesis correlated with surgical management. J Bone Joint Surg Am 41-A: 635–664. 13664703

[28] Ratkaj I, Bujak M, Jurisic D, Loncar MB, Bendelja K, et al (2012) Microarray Analysis of Dupuytren's Disease Cells: The Profibrogenic Role of the TGF-beta Inducible p38 MAPK Pathway. Cellular Physiology and Biochemistry 30: 927–942. 22965824 10.1159/000341470

[29] Verjee LS, Verhoekx JSN, Chan JKK, Krausgruber T, Nicolaidou V, et al (2013) Unraveling the signaling pathways promoting fibrosis in Dupuytren's disease reveals TNF as a therapeutic target. Proceedings of the National Academy of Sciences of the United States of America 110: E928–E937. 23431165 10.1073/pnas.1301100110 PMC3593900

[30] Rehman S, Salway F, Stanley JK, Ollier WE, Day P, et al (2008) Molecular phenotypic descriptors of Dupuytren's disease defined using informatics analysis of the transcriptome. J Hand Surg Am 33: 359–372. 18343292 10.1016/j.jhsa.2007.11.010

[31] Millesi H, Reihsner R, Hamilton G, Mallinger R, Menzel EJ (1995) Biomechanical properties of normal tendons, normal palmar aponeuroses, and tissues from patients with Dupuytren's disease subjected to elastase and chondroitinase treatment. Clin Biomech (Bristol, Avon) 10: 29–35. 10.1016/0268-0033(95)90434-b 11415528

[32] Lam WL, Rawlins JM, Karoo RO, Naylor I, Sharpe DT (2010) Re-visiting Luck's classification: a histological analysis of Dupuytren's disease. J Hand Surg Eur Vol 35: 312–317. 20181770 10.1177/1753193410362848

[33] Ricard-Blum S (2011) The Collagen Family. Cold Spring Harbor Perspectives in Biology 3.. 10.1101/cshperspect.a004978 PMC3003457 21421911

[34] Plumb DA, Dhir V, Mironov A, Ferrara L, Poulsom R, et al (2007) Collagen XXVII is developmentally regulated and forms thin fibrillar structures distinct from those of classical vertebrate fibrillar collagens. Journal of Biological Chemistry 282: 12791–12795. 17331945 10.1074/jbc.C700021200 PMC2688011

[35] Wenstrup RJ, Florer JB, Brunskill EW, Bell SM, Chervoneva I, et al (2004) Type V collagen controls the initiation of collagen fibril assembly. Journal of Biological Chemistry 279: 53331–53337. 15383546 10.1074/jbc.M409622200

[36] Ansorge HL, Meng XM, Zhang GY, Veit G, Sun M, et al (2009) Type XIV Collagen Regulates Fibrillogenesis PREMATURE COLLAGEN FIBRIL GROWTH AND TISSUE DYSFUNCTION IN NULL MICE. Journal of Biological Chemistry 284: 8427–8438. 19136672 10.1074/jbc.M805582200 PMC2659201

[37] Kreis T, Vale R (1999) Guidebook to the Extracellular Matrix, Anchor, and Adhesion Proteins.

[38] Couchman JR, Pataki CA (2012) An introduction to proteoglycans and their localization. J Histochem Cytochem 60: 885–897. 23019015 10.1369/0022155412464638 PMC3527888

[39] Reed CC, Iozzo RV (2002) The role of decorin in collagen fibrillogenesis and skin homeostasis. Glycoconj J 19: 249–255. 12975602 10.1023/A:1025383913444

[40] Landolt RM, Vaughan L, Winterhalter KH, Zimmermann DR (1995) Versican is selectively expressed in embryonic tissues that act as barriers to neural crest cell migration and axon outgrowth. Development 121: 2303–2312. 7671797 10.1242/dev.121.8.2303

[41] Zhang Y, Cao L, Yang BL, Yang BB (1998) The G3 domain of versican enhances cell proliferation via epidermial growth factor-like motifs. J Biol Chem 273: 21342–21351. 9694895 10.1074/jbc.273.33.21342

[42] Ekblom P, Timpl R (1996) Laminins: Taylor & Francis.

[43] Berndt A, Kosmehl H, Katenkamp D, Tauchmann V (1994) Appearance of the myofibroblastic phenotype in Dupuytren's disease is associated with a fibronectin, laminin, collagen type IV and tenascin extracellular matrix. Pathobiology 62: 55–58. 7524526 10.1159/000163879

[44] Garg M, Kanojia D, Okamoto R, Jain S, Madan V, et al (2014) Laminin-5gamma-2 (LAMC2) is highly expressed in anaplastic thyroid carcinoma and is associated with tumor progression, migration, and invasion by modulating signaling of EGFR. J Clin Endocrinol Metab 99: E62–72. 24170107 10.1210/jc.2013-2994 PMC3879679

[45] Pasquali-Ronchetti I, Guerra D, Baccarani-Contri M, Fornieri C, Mori G, et al (1993) A clinical, ultrastructural and immunochemical study of Dupuytren's disease. J Hand Surg Br 18: 262–269. 8501391 10.1016/0266-7681(93)90125-y

[46] Freeman SD, Moore WM, Guiral EC, Holme AD, Turnbull JE, et al (2008) Extracellular regulation of developmental cell signaling by XtSulf1. Developmental Biology 320: 436–445. 18617162 10.1016/j.ydbio.2008.05.554

